# The Role of PD-1 in Acute and Chronic Infection

**DOI:** 10.3389/fimmu.2020.00487

**Published:** 2020-03-24

**Authors:** Jil M. Jubel, Zachary R. Barbati, Christof Burger, Dieter C. Wirtz, Frank A. Schildberg

**Affiliations:** ^1^Clinic for Orthopedics and Trauma Surgery, University Hospital Bonn, Bonn, Germany; ^2^Tufts University School of Medicine, Boston, MA, United States

**Keywords:** PD-1, PD-L1, PD-L2, T cell exhaustion, acute infection, chronic infection, checkpoint inhibitor, infectious disease

## Abstract

PD-1 as an immune checkpoint molecule down-regulates T cell activity during immune responses in order to prevent autoimmune tissue damage. In chronic infections or tumors, lasting antigen-exposure leads to permanent PD-1 expression that can limit immune-mediated clearance of pathogens or degenerated cells. Blocking PD-1 can enhance T cell function; in cancer treatment PD-1 blockade is already used as a successful therapy. However, the role of PD-1 expression and blocking in the context of acute and chronic infections is less defined. Building on its success in cancer therapy leads to the hypothesis that blocking PD-1 in infectious diseases is also beneficial in acute or chronic infections. This review will focus on the role of PD-1 expression in acute and chronic infections with virus, bacteria, and parasites, with a particular focus on recent studies regarding PD-1 blockade in infectious diseases.

## Introduction

T lymphocytes play an important role both in defending the body against pathogenic microbes as well as maintaining tolerance to self. These antagonistic functions are orchestrated by different T cell subsets, which include the effector T cells (T_eff_), such as cytotoxic T cells (CTLs) or helper T cells (T_h_), and regulatory T cells (T_reg_). Effector T cells play an important role in acquired immunity, whereas regulatory T cells are pivotal for the development of tolerance to self and attenuating the immune response in order to reduce collateral tissue damage at the site of inflammation. As a consequence, the dynamic ratios of various T cell subsets (e.g., the T_eff_:T_reg_ ratio) are important for maintaining effective immunity and tolerance to self (1). The antagonistic functions are orchestrated by several costimulatory receptors that provide stimulatory (costimulatory) and inhibitory (coinhibitory) signals to T cells, thereby modulating T cell activation and effector responses to foreign or self-antigen. Signaling through costimulatory and coinhibitory receptors only occurs secondary to successful ligation of the T cell receptor (TCR) with its cognate major histocompatibility complex (MHC). Thus, costimulation has become regarded as a second signal to T cells, providing them with additional information about the local microenvironment and inflammatory state of the host.

**Figure 1:**
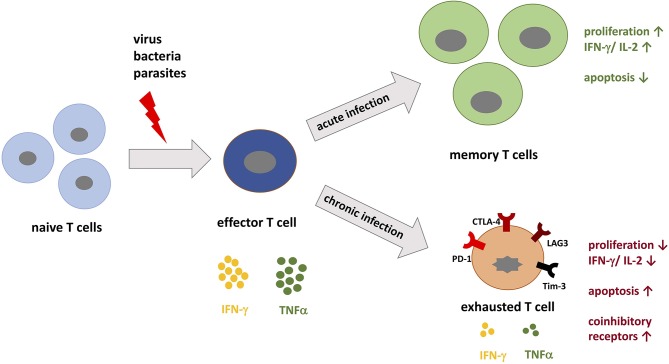
T cell differentiation during acute vs. chronic infections. When pathogenic peptides are presented by major histocompatibility complex (MHC) to naive T cells during infection, corresponding T cells are activated and differentiate into effector T cells specific for the pathogen. During acute infection, the immune system is able to specifically target the pathogen and eradicate it. During this process, memory T cells develop and confer immunological memory against recurrent infection. In contrast, during chronic infection the persistent load of pathogenic peptides leads to permanent stimulation of T cells, which promotes T cell exhaustion. T cell exhaustion is in part defined by the upregulation of coinhibitory receptors such as PD-1, LAG-3, and Tim-3. These receptors inhibit T cells by decreasing IL-2 production, proliferation, and the threshold for apoptosis.

During infection, peptide antigens from microbes are presented on MHC complexes (peptide-MHC) to naive T cells by professional antigen-presenting cells (APCs). Importantly, in order for T cells to recognize their cognate peptide-MHC complex, they express one of two co-receptors, CD4 or CD8. CD4^+^ T cells recognize MHC class II molecules and are responsible for orchestrating the immune response against extracellular pathogens, such as bacteria or helminths, by providing “help” to B cells and by activating innate effector cells, such as macrophages and NK cells. In contrast, CD8^+^ T cells recognize MHC class I molecules and function as cytotoxic cells that target virally infected or malignant cells. After a T cell receives both primary and secondary signals via the TCR:MHC complex and through costimulation, the T cell is now considered activated and can differentiate into effector-type and memory cells (Figure 1). Costimulatory and coinhibitory receptors expressed on the surface of T cells participate in modulating T cell differentiation and thus the immune response [reviewed by (2)]. Signaling through these receptors provides information to the T cell about the local environment as well as alter T cell survival, differentiation potential, and metabolism (3–7). The coinhibitory receptor Programmed Cell Death-1 (PD-1) plays an important immunoregulatory role by reducing initial T cell activation, fine-tuning T cell differentiation and effector functions, and by contributing to the development of immunological memory (8–11). The inhibitory effects of PD-1 are mediated by engagement with its ligands PD-L1 and PD-L2 (12, 13). It is accepted that PD-1 signaling through T cells limits immune-mediated tissue damage during infection. Seeing as the PD-1 pathway also serves to limit the activation of self-reactive T cells, this signaling pathway plays a role in reducing the risk for autoimmunity and immunopathology (11).

Given the immunoregulatory role that PD-1 plays during inflammation, both foreign microorganisms and transformed host cells have evolved mechanisms to exploit the PD-1 pathway to evade host defenses (14, 15). Thus, examining PD-1 pathway during infection in both mice and humans has been an area of intensive research. In this review, we will cover what is known about the role of the PD-1 pathway during infectious disease. While the PD-1 pathway is an important player in regulating the activation and function of T cells at numerous points during T cell development, activation, and effector phases, here we will focus primarily on the role that the PD-1 pathway has on T cell effector responses during infection. We will also comment on the therapeutic role that PD-1 blockade may have in the context of acute and chronic infection with pathogens that are resistant to treatment. The term PD-1 blockade is a broad term that includes various mechanisms of pathway inhibition and will be used herein after to describe the use of either blocking antibodies against PD-1 or PD-L1, unless otherwise specified. Specifically, we will summarize what is known about the PD-1 pathway in the context of both acute and chronic viral infections, bacterial infections, and comment on its role during sepsis.

## What Is PD-1?

PD-1 exists as a monomeric surface glycoprotein and can be recruited to the TCR signalosome during TCR ligation with peptide-MHC complexes (16, 17). It is encoded by the *PDCD*1 gene, which contains five exons. Exon 1 encodes a signal sequence, exon 2 encodes an IgV-like domain, and exon 3 encodes a stalk and transmembrane domain. Exons 4 and 5 encode the cytoplasmic domain, which together contain an immunoreceptor tyrosine-based inhibitory motif (ITIM) and an immunoreceptor tyrosine-based switch motif (ITSM) (18, 19). PD-1 is inducibly expressed on CD4^+^ and CD8^+^ T cells, natural killer (NK) cells, natural killer T (NKT) cells, B cells, macrophages, and some dendritic cell (DC) subsets. T cell receptor (TCR) signaling and some cytokines, such as interleukin-2 (IL-2), interleukin-7 (IL-7), and type I interferons (IFNs), can all induce PD-1 expression on T cells (20). PD-1 expression is specifically upregulated on activated T cells and its expression can be upregulated within 24 h depending on the strength or concentration of the aforementioned stimuli. Thus, PD-1 can serve as a marker of T cell activation both *in vitro* and *in vivo*. The kinetics of PD-1 expression vary between acute and chronic infection. For example, PD-1 expression on antigen-specific T cells is transient in the context of acute infection (i.e., there is a temporary antigen load). However, in the context of chronic infection (i.e., a continuous antigen burden), PD-1 expression is sustained (21, 22). Continuous PD-1 expression on antigen-specific T cells in the context of chronic inflammation positively correlates with T cell dysfunction (21–23). These dysfunctional T cells are said to be “exhausted” (Figure 1). However, studies have found that PD-1 is necessary for the reinvigoration of exhausted T cells. Moreover, the absence of PD-1 leads to terminally exhausted T cells that cannot be recovered (14, 24). Thus, PD-1 helps avert the terminal exhaustion of T cells. Exhausted T (T_ex_) cells represent a heterogeneous population of antigen-specific T cells, whereby the expression of additional coinhibitory receptors and unique transcriptomes contribute to their overall dysfunctional state (25). The varying levels of other coinhibitory receptors further act to modulate T cell function in the context of acute and chronic infections (22).

The inhibitory effects of PD-1 are mediated by engagement with its ligands PD-L1 and PD-L2 (8, 12). While PD-L2 is inducibly expressed by dendritic cells (DCs), macrophages, and cultured bone marrow-derived mast cells (2), PD-L1 is constitutively expressed on the aforementioned cell types, B cells, and mesenchymal stem cells (26). Additionally, PD-L1 is expressed by a variety of non-hematopoietic cells, such as hepatocytes, vascular endothelial cells, epithelial cells, myocytes, pancreatic islet cells, and astrocytes. Of note, there is a remarkable degree of PD-L1 expression at sites of immune privilege, such as the placenta and the eye (27, 28). While there is overlap between PD-1 activation via PD-L1 and PD-L2, there is evidence to support that they independently regulate T cell responses. For example, research supports PD-L2 as the predominant regulator of CD8^+^ T cell proliferation and for PD-L1 as an important player for the induction of peripheral T_reg_ cells (see below) (25, 29).

**Figure 2:**
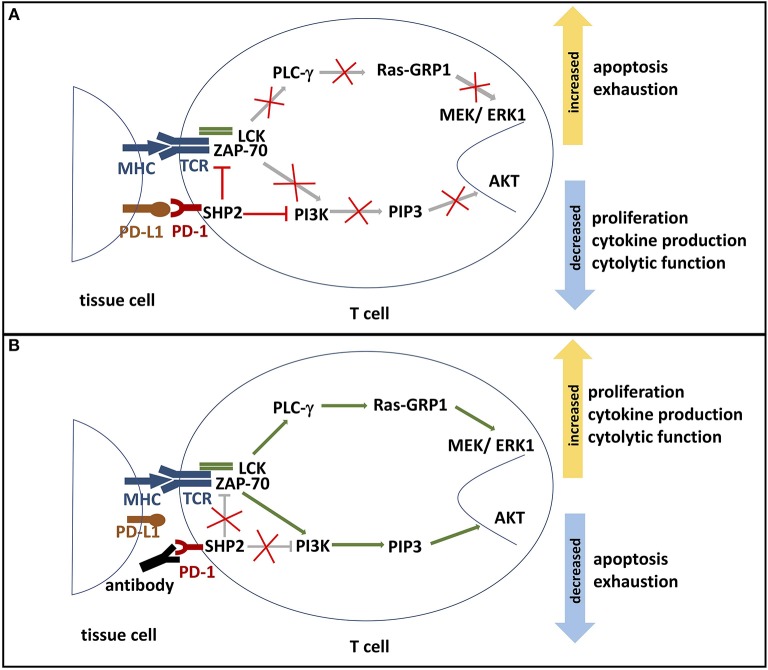
**(A)** PD-1 signaling pathway. The binding of PD-L1 or PD-L2 to its receptor PD-1 results in the phosphorylation of PD-1's ITSM and ITIM tyrosine motifs, which are located on its cytoplasmic domain. Phosphorylation leads to the recruitment of protein tyrosine phosphatases, such as SHP2. SHP2 subsequently inhibits two important pathways: One, it competes with kinases to prevent the activation of PI3K by phosphorylation. This inhibits phosphorylation of PIP2 to PIP3, thereby inhibiting Akt activation. Deactivation of serine-threonine kinase Akt reduces T cell proliferation, increases apoptosis, and promotes T cell exhaustion. Effector functions such as cytokine production and cytolytic function are also reduced. Two, SHP2 inhibits the Ras-MEK-ERK pathway. Dephosphorylation of ZAP-70 and LCK antagonize the positive downstream effects of the MHC-TCR pathway, leading to deactivation of PLC-γ, Ras-GRP1 and MEK/ERK1. ERK1 normally activates transcription factors that induce T cell proliferation and differentiation. Thus, decreased ERK1 activation reduces proliferation and differentiation potential. **(B)** Blockade of PD-1. In the presence of a PD-1 blocking antibody, the engagement of PD-1 and its ligands is inhibited. Consequently, SHP2 is not activated and neither PI3K/Akt pathway nor Ras-MEK-ERK pathway are repressed. Activated AKT and ERK support T cell cytokine production, proliferation, and differentiation. Furthermore, PD-1 blockade reduces T cell exhaustion and the rate of apoptosis. ITSM, immunoreceptor tyrosine-based switch motif; ITIM: immunoreceptor tyrosine-based inhibition motif; SHP2, Src homology region 2 domain-containing phosphatase 2; PI3K, phosphoinositide 3-kinase; PIP2, phosphoinositide-3,4-bisphosphate; PIP3, phosphatidylinositol-3,4,5-trisphosphate; Ras, rat sarcoma; MEK, MAK-/ERK-kinase; ERK1, extracellular-signal regulated kinases 1; Zap-70, zeta-chain-associated protein kinase 70; LCK, lymphocyte-specific protein tyrosine kinase; PLC-γ, Phosphoinositide phospholipase C-γ.

PD-1 modulates T cell activity by a number of mechanisms. Engagement of PD-1 with PD-1 ligands has been shown to inhibit proximal TCR signaling and downstream responses (Figure 2). Data collected from *in vitro* studies suggest that PD-1 engagement accomplishes this regulatory activity by multiple mechanisms. Engagement of PD-1 ligands with PD-1 leads to tyrosine phosphorylation of the cytoplasmic tail of PD-1 and the subsequent recruitment of the phosphatase SHP-2, a protein tyrosine phosphatase (PTP). PTPs function to dephosphorylate kinases and as a consequence, the positive signals downstream TCR and CD28 activation become antagonized. SHP-2 has been shown to directly attenuate TCR signaling by reducing phosphorylation of the Zap70/CD3ε signalosome (11, 30, 31). The downstream effects of PD-1 signaling include inhibition of AKT, phosphoinositide 3-kinase (PI3K), extracellular-signal regulated kinase (ERK), and phosphoinositide phospholipase C-γ (PLCγ) and regulation of the cell cycle leading to decreased IFN-γ/IL-2 production, reduced proliferation potential, and increased risk for apoptosis (3, 16, 26, 31). Additionally, PD-1 signaling alters T cell metabolism by inhibiting glycolysis and by promoting lipolysis and fatty acid oxidation (32, 33).

Together, the downstream effect of PD-1 signaling serves to modulate T cell activation and effector function in the context of infection. Murine models of PD-1 deficiency are associated with lethal immunopathology during acute infection. Immunopathology is associated with high levels of systemic cytokines, endothelial cell death, and local tissue damage (21, 34). These data support the role for the PD-1 pathway in limiting the pro-inflammatory immune response during infection and suggest that the PD-1 pathway contributes to immune cell contraction after infection. Additionally, the PD-1 pathway plays an important role in regulating tolerance to self. In murine models, blocking the PD-1 pathway via genetic knock-down or through the administration of blocking antibodies increases the risk for developing autoimmune dilated cardiomyopathy and experimental autoimmune encephalomyelitis (35). Additionally, transgenic mice that express PD-1 with a mutant ITIM motif develop lupus-like autoimmune diseases (36, 37). In humans, single-nucleotide polymorphisms (SNP) of the *PDCD1* gene have been connected with different autoimmune diseases, such as systemic lupus erythematosus (38). Whether SNPs are correlative or causative is not yet identified (11).

Inhibitory signals, like PD-L1 and PD-L2, control induction and maintenance of tolerance to self-antigens through the PD-1 pathway (12, 37). One mechanism by which the PD-1 pathway may regulate auto-reactivity is through the induction of regulatory T (T_reg_) cells in peripheral circulation. This is in contrast to natural T_reg_ cells, which are centrally derived through thymic selection and express the transcription factor forkhead box P3 (FoxP3), a key transcriptional regulator of the T_reg_ cell phenotype. Naive CD4^+^ T cells that enter peripheral circulation can be induced to express FoxP3 through the engagement of both the TCR and PD-1 on its surface (38). Both natural and induced T_reg_ cells function to suppress the immune response by producing immune modulatory molecules, such as the anti-inflammatory cytokines transforming growth factor-β (TGF-β) and interleukin-10 (IL-10) (15, 39). Activated T_reg_ cells express higher levels of PD-1, which is thought to contribute to T_reg_ cell activity. Consistent with this, blocking PD-1 leads to decreased suppressive activity of T_reg_ cells *in vivo* (26). The induction of peripheral T_reg_ cells is thought to reduce both the activation and effector activity of self-reactive CD4^+^ and CD8^+^ T cells. Together, these data underscore the complexity of PD-1 engagement and the unique roles that the PD-1 pathway plays in the context of infection and autoimmunity. It is thus thought that the PD-1 pathway works to reduce the activation of self-reactive T cells as well as modulate the degree of activation and the effector activity of antigen-specific T cells.

The role of inhibiting the PD-1 pathway has had profound impact on tumor biology and cancer immunobiology. PD-L1 expressing tumor cells are able to escape both detection and elimination by tumor-specific CD8^+^ T cells (40, 41). Furthermore, PD-L1 expressing tumor cells both induce T cell exhaustion of tumor-specific T cells as well as induce tumor-specific T_reg_ cells (42, 43). The impact of PD-1 blockade is so profound that it has already been translated to therapy to treat some forms of cancer despite the pathway's recent discovery. PD-1 blockade is an example of checkpoint inhibition, whereby natural immune checkpoints are therapeutically inhibited thereby permitting disease-specific T cell activity (11). Monoclonal blocking antibodies against PD-1 and PD-L1 are now used to treat advanced melanoma, non-small cell lung cancer, and head and neck squamous cell cancer (44). Checkpoint blockade increases the effector function of tumor-specific CD8^+^ T cells, which exert powerful natural defenses against some forms of malignancy. Where PD-1 blockade alone is not sufficient, combination therapies targeting more than one checkpoint have proven to reinvigorate tumor-specific T cells and reduce or even completely eliminate the tumor burden in some patients (29). Despite these promising results, it's important to note that some patients developed severe immune-related adverse events, such as colitis, hepatitis and skin disorders. These results underscore the need to better understand why some patients benefit and other patients suffer from checkpoint blockade (11, 29). Interestingly, the impact that PD-1 blockade has on viral and bacterial infection is less well-defined. Here, we will give an overview of what is known about PD-1 blockade in the context of acute and chronic infections caused by notable pathogens.

## What Is Known About the PD-1 Pathway in Chronic Viral Infection?

During the course of a chronic viral infection there is a continuous burden of viral antigen. As a consequence, antigen-specific T cells are continually stimulated during the course of infection. This causes antigen-specific T cells to enter a state called T cell exhaustion (21–23). Exhausted T cells loose effector functions, such as reduced production of IL-2 and other cytokines and a reduction in proliferative and cytotoxic capacity (Figure 1). Another important indicator of T cell exhaustion is the upregulation of additional coinhibitory receptors (1, 29). The phenotypic characterization of T_ex_ cells was born out of murine studies aimed at understanding the differences between T cell responses during acute and chronic infections with lymphocytic choriomeningitis virus (LCMV). In these seminal experiments, antigen-specific T cells taken from mice infected with a chronic strain of LCMV exhibited higher and more persistent levels of PD-1 expression when compared to antigen-specific T cells taken from mice infected with an acute strain of LCMV (22). Two different populations of antigen-specific dysfunctional T cells were identified by their expression level of PD-1. In the population of T cells where PD-1 expression was intermediate, T cell effector function could be recovered by blocking PD-1. This is in contrast to the population of T cells that expressed high levels of PD-1. PD-1 blockade could not rescue effector function of these T cells and thus were considered to be terminally exhausted (14, 45). Importantly, Blackburn et al. demonstrate that blocking the PD-1 pathway could functionally reverse T cell exhaustion and improve control over viral infection by increased T cell proliferation, killing capacity of CD8^+^ T cells and cytokine production (11, 22). This finding demonstrated an important role for PD-1 signaling in restraining ongoing responses in the context of chronic or persistent viral infection (15, 21). Combined blockade of both PD-1 and the coinhibitory molecule LAG-3 provided even better results when compared to blocking PD-1 alone, supporting the role of additional coinhibitory molecules in T cell exhaustion (15).

In humans, PD-1 expression on antigen-specific T cells and T cell exhaustion have been documented in numerous chronic infections, including infections with the hepatitis B virus (HBV), hepatitis C virus (HCV), and human immunodeficiency virus (HIV) (46–48). There are an estimated 257 million people chronically infected with HBV and more than 800,000 people die each year due to complications such as cirrhosis and liver cancer (49). While hepatitis B is easily prevented by vaccination, established infections are often resistant to treatment, thereby leaving infected individuals on life-long antiviral treatment. In chronic infection with HBV, PD-1 expression on HBV-specific T cells is increased (46, 47, 50, 51). Not surprisingly, in these studies PD-1 expression negatively correlates with CD8^+^ T cell responses (52). Research also suggests that the expression of PD-1 on HBV-specific CD8^+^ T cells at the earliest time points of infection positively correlate with serum ALT levels, thereby suggesting that early PD-1 expression in patients with chronic hepatitis B may serve as a biomarker for liver damage (53, 54). Newer studies also demonstrate similar results when examining PD-1 expression on HBV-specific CD4^+^ T cells (54).

Efforts have been made to examine how blocking the PD-1 pathway would impact the course of the infectious disease. The results and efficacy of PD-1 blockade vary considerably across studies and between pathogens (Table 1). In the context of chronic infection with HBV, PD-1 blockade increases T cell proliferation and the production of interferon-γ (IFN-γ) and IL-2 by HBV-specific CD8^+^ T cells taken from both the peripheral blood and livers of patients with chronic hepatitis B (55). While PD-1 blockade restored CD8^+^ T cell activity in this study, another study using HBV-specific T cells taken from the peripheral blood of chronic hepatitis B paints a different picture. Tang et al. demonstrate that blocking the PD-1 pathway more significantly altered CD4^+^ T cell function rather than CD8^+^ T cell function. Specifically, they report enhanced IFN-γ, IL-2 and TNFα expression by treated CD4^+^ T cells when compared to CD4^+^ T cells treated with an isotype control, as well as increased expression of the transcription factor T-bet and reduced expression of the transcription factor GATA-3 (54, 56). Differences in studies as such could potentially be resolved by better defining the HBV-specific T cell population. For example, one could imagine different responses to PD-1 blockade when more than one population of exhausted antigen-specific T cells exist (e.g., one population that expresses multiple coinhibitory receptors and a second population that predominantly expresses PD-1). Furthermore, differences in the virulence of the viral strain and variability between the populations being studied may also contribute to the degree of T cell exhaustion in the context of a chronic infection. Indeed, Tang et al. report that HBV-specific T cells express additional coinhibitory receptors, for example Tim-3 and LAG-3 (54, 71) in the context of chronic viral infection. It's plausible that PD-1 blockade may exert different effects on the CD4^+^ and CD8^+^ T cell compartments depending on the time of treatment (early vs. late) or on the degree of T cell exhaustion as measured by the number of coinhibitory receptors expressed by HBV-specific T cells. Using combination therapy to block two or more coinhibitory pathways may better serve T cell exhaustion in the context of chronic infection with HBV. However, additional work is needed to prove the efficacy of combination therapy. Importantly, while checkpoint inhibition might enhance viral clearance in patients with chronic hepatitis B, there is a risk that reactivation of subsets of exhausted T cells may increase tissue immunopathology (72).

**Table 1:** Summary of PD-1 blockade in various infectious diseases.

**Type of infection**	**Pathogen**	**Target species**	**Affected cells**	**Results**	**References**
Chronic viral infection	HBV	Human	CD4^+^, CD8^+^	**Anti-PD-L1 treatment** IFN-γ + IL-2 + TNFα ↑ IL-10 ↓ Proliferation ↑ Exhaustion ↓	(54–56)
	HCV	Human	CD8^+^	**Anti-PD-L1** **+** **anti-CTLA-4 treatment** IFN-γ + TNFα ↑ Effector function ↑ Proliferation ↑ Viremia ↓	(57, 58)
	HIV	Human, mouse	CD4^+^, CD8^+^, naive + memory T cells	**Anti-PD-L1 treatment** IFN-γ + IL-13, IL-21 ↑ Cytokine production ↑ Proliferation ↑ Expansion ↑ HIV latency reversal ↑ Viremia ↓	(59–62)
	SIV	Monkey	CD8^+^, memory B cells	**Anti-PD-1 treatment** IFN-γ + IL-2 + TNFα ↑ Effector function ↑ Proliferation ↑ Expansion ↑ Survival rate↑ Viremia ↓	(63)
Acute viral infection	HMPV	Mouse	CD8^+^	**Anti-PD-L1/L2 treatment** IFN-γ + TNFα + IL-6 ↑ Pulmonary effector function ↑ Lung viral titers ↓ *neg.:* higher degree of airway dysfunction	(64)
	Rabies virus	Mouse	CD8^+^	**Anti-PD-L1 treatment** Immune response ↑ Survival rate ↑	(65)
	Influenza	Mouse	CD8^+^	**Anti-PD-L1 treatment** IFN-γ + granzyme B ↑ Viremia ↓	(64, 66)
Bacterial	H. pylori	Human	CD4^+^	**Anti-PD-L1 treatment** IL-2 ↑ Proliferation ↑	(67)
Parasitic	T. gondii	Mouse	CD8^+^	**Anti-PD-L1 treatment** IFN-γ + granzyme B ↑ Effector function ↑ Mortality ↓	(68)
	L. infantum	Dog	Lymphocyte	**Anti-PD-1 treatment** IFN-γ + NO ↑ IL-4 + IL-10 ↓ Superoxide formation ↑	(69)
	L. donovani	Mouse	CD4^+^, CD8^+^ APC	**Anti-PD-L1 treatment** IFN-γ + TNFα ↑ Proliferation ↑ Parasite burden ↓ APC: T cell priming function ↑	(70)

*Summary of the most important anti-PD-1/PD-L1 therapies in various infections mentioned in this review article. PD-1, programmed cell death protein 1; PD-L1, programmed cell death ligand 1; HBV, hepatitis B virus; HCV, hepatitis C virus; HIV, human immunodeficiency virus; SIV, simian immunodeficiency virus; HMPV, human metapneumovirus; H. pylori, helicobacter pylori; T. gondii, toxoplasma gondii; L. infantum/donovani, leishmania infantum/donovani; IFN-γ, interferon gamma; TNFα, tumor necrosis factor α; IL, interleukin; NO, nitric oxide; APC, antigen-presenting cells*.

Studies examining patients with chronic hepatitis C virus (HCV) also have varying results. HCV-specific CD8^+^ T cells have been shown to express PD-1 in several studies (57, 58, 73, 74). *Ex vivo* analysis of CD8^+^ T cells taken from patients with both acute and chronic HCV show that PD-1 expression on HCV-specific CD8^+^ T cells directly correlates with functional impairment. Interestingly, data from this same study reveal that blocking the PD-1 pathway restores the effector function of peripheral HCV-specific CD8^+^ T cells but not on HCV-specific CD8^+^ T cells taken from the liver (58). Importantly, cells taken from the liver were more highly positive for PD-1 than those taken from the periphery, suggesting that these cells may be more “exhausted” than those circulating in the periphery (58). These data point to differences in compartmentalization and the tissue microenvironment being important factors to consider when using PD-1 expression as a marker for T cell exhaustion and when predicting responsiveness to PD-1 blocking therapy. These results support the need to better define populations of antigen-specific T cells in the context of chronic infection when considering the impact that PD-1 blockade could have on the course of an infection (75, 76). Indeed, Gardiner et al. address the fact that the degree of T cell exhaustion (i.e., the degree of PD-1 expression and the number of additional coinhibitory receptors expressed) may confound the efficacy of PD-1 blockade. *Ex vivo* experiments done on PD-1^+^ HCV-specific CD8 T cells taken from the livers of patients chronically infected with HCV show that combined blockade against CTLA-4 and PD-1 restored T cell function whereas blockade against PD-1 or CTLA-4 alone did not (57) (Table 1).

In a randomized, double-blind, placebo-controlled study assessing the efficacy of a single dose of anti-PD-1 monoclonal antibody for the treatment of chronic hepatitis C in human patients, a protocol-defined clinical response (HCV RNA reductions of more than 0.5 log_10_ IU/mL were observed on 2 or more consecutive visits) was observed in five out of 45 patients. Three out of 20 patients treated with the highest administered dose (10 mg/kg) experienced >4 log_10_ IU/mL reductions in HCV RNA at the end of a 12-weeks, and two patients achieved HCV RNA below the lower limit of quantitation (74). While these data are promising, these numbers may not be suitable for justifying the cost of PD-1 blockade for the treatment of chronic hepatitis C given the efficacy of direct acting antivirals (e.g., Sofosbuvir or Ledipasvir) for treating both acute and chronic infections (77, 78). However, these data provide us with an important proof of concept that warrants further investigation for the use of PD-1 blockade to treat chronic infections. In line with the work done in the context of chronic hepatitis B, it is important for investigators to better define the T cell populations that respond to PD-1 blocking therapy in order to establish criteria that can be used to target patients that may benefit from immunotherapy.

Infection with HIV has a significant burden on the world population. Worldwide, there are over 37.9 million HIV-infected people and 1 million people die each year due to complications (79). Dysfunctional HIV-specific T and B cell responses are one of the primary reasons why patients living with HIV exhibit diminished immune control over the course of infection (80). Chronic HIV infection is characterized by continuous viral replication in the majority of HIV infected persons, which if left untreated, invariably leads to disease progression and acquired immunodeficiency syndrome (AIDS). Exceptions do exist; elite controllers are individuals that can control viral infection in the absence of therapy. Unsurprisingly, PD-1 expression on HIV-specific CD4^+^ and CD8^+^ T cells is associated with T cell exhaustion and disease progression (59, 81, 82). Additionally, in comparison to healthy controls, HIV-infected individuals have a higher frequency of T_reg_ cells (83–85). PD-1 expression correlated positively with additional markers of disease progression such as viral load and reduced CD4^+^ T cell numbers (86). While today's antiretroviral therapy (ART) permits individuals living with HIV to lead relatively normal and healthful lives, these drugs do not eliminate the virus and patients must remain on antiretroviral therapy for the rest of their lives (11).

CD4^+^ T cells constitute the major cell type infected by HIV. The death and loss of functional CD4^+^ T cells causes AIDS in these individuals. Immuno-phenotyping CD4^+^ T cells taken from HIV-positive patients receiving suppressive ART reveals that these cells express PD-1, TIGIT and LAG-3 alone or in combination (48, 59, 87, 88). The population of CD4^+^ T cells that coexpressed all three coinhibitory receptors positively correlated both with the size of the HIV reservoir and the amount of integrated HIV DNA. These authors also demonstrate that memory CD4^+^ T cells expressing coinhibitory receptors contribute to the pool of HIV genomes during ART. Thus, it's possible that blocking coinhibitory receptors, such as PD-1, may target latently infected cells in virally suppressed individuals and possibly facilitate viral eradication (60, 72, 88). Indeed, *ex vivo* studies performed on CD4^+^ T cells taken from ART-suppressed patients treated with PD-1 blockade potentiates HIV latency reversal. Importantly, another study has revealed that in both aviremic and ART-suppressed patients the most important source of virus comes from PD-1^+^ T_h_ cells (60, 72).

It has been shown that the number of HIV-specific CD4^+^ T cells correlate with control of viremia (89). This is consistent with data that has shown that the depletion of CD4^+^ T cells leads to a loss of CTL activity. The loss of CTL activity is caused most directly be the loss of CD4^+^ T helper cells and their many subsets (e.g., T_h_1, T_h_2, and T follicular cells). Additional work has shown that *in vitro* blockade of the PD-1 pathway restores HIV-specific CD4^+^ T cell proliferation and enhances HIV-specific CD4^+^ T cells ability to produce T helper cell subset specific cytokines, such as IFN-γ (T_h_1), IL-13 (T_h_2), and IL-21 (TFH) (59) (Table 1). Blockade also restored cytokine secretion in both CD4^+^ T cells that expressed intermediate and high levels of PD-1. When comparing HIV-specific CD4^+^ and CD8^+^ T cells, CD4^+^ T cells expressed lower levels of the inhibitory receptors CD160 and 2B4, thereby demonstrating key differences in immunoregulation between CD4^+^ and CD8^+^ T cells during chronic infection with HIV (59, 90). Together, these data show that the PD-1 pathway impairs multiple subsets of HIV-specific T helper cell responses (90). Studies performed in humanized BLT mice infected with HIV report similar results. Mice treated with PD-1 blockade exhibit improved CD4^+^ T cell levels and reduced viremia when compared to controls (61) (Table 1).

PD-1 blockade has also been shown to have an impact on antigen-specific CD8^+^ T cells. In similar experiments using humanized BLT mice, HIV-infected mice treated with PD-1 pathway blocking antibodies reduced viremia by a 45-fold reduction when compared to control animals 4-weeks post-treatment. The treatment group also exhibited an increase in the number of HIV-specific CD8^+^ T cells (62). Investigation of PD-1 blockade in the context of chronic simian immunodeficiency virus (SIV) in macaque monkeys yielded similar results. In these experiments, the authors demonstrate that PD-1 blockade causes SIV-specific CD8^+^ T cells to respond by both rapid expansion and improved functionality. Importantly, the authors investigated responses both in the blood and the gut and saw enhanced T cell immunity in both compartments. The investigators also measured improved memory B cell responses and increased levels of SIV specific antibody. Treated animals exhibited significant reductions in viremia and prolonged survival when compared to untreated animals (63) (Table 1). Despite these promising results, one should be aware of the systemic effects of the immunotherapy treatments like PD-1 blockade. At present, there is one case of HBV reactivation in a HIV-infected patient treated with a PD-1 inhibitor (76).

Given these data, it has been postulated that impaired CD4^+^ T cell help to B cells and B cell dysfunction may help explain the low frequency of broadly neutralizing antibodies in HIV-infected individuals. Additional work has also been done to investigate the level of expression of PD-1 on the surface on virus-specific B cells during chronic infection with HIV. These data demonstrate that during chronic infection with HIV, virus-specific B cells respond to antigen poorly and that this poor response can be relieved by suppressing the PD-1 pathway (91). In addition, there is evidence that exhausted B cells in HIV express other coinhibitory receptors. Blocking these receptors in addition to PD-1 could enhance the immune response (15). Additional work also suggests that chronic infection with HIV also dampens the activity of T follicular helper cells (TFH) (70), which are necessary for the proper formation of germinal centers that facilitate the development of high-affinity antibodies by B cells (92). Other studies have reported, that in highly viremic HIV infected children the rate of memory TFH was lower compared to controls. It was shown, that a high rate of memory TFH was associated with elevated ratio of resting memory B cells (93). Together, these results demonstrate that blocking the PD-1 pathway can enhance both the cellular and humoral immune response during infection with pathogenic immunodeficiency viruses (15). Despite these promising results, one should be aware of the other coinhibitory receptors that play an important role during chronic infections. More research is needed to understand the correlation of all these receptors and their different roles in T cell exhaustion.

## Acute Viral Infection and the PD-1 Pathway

The pathobiology of acute viral infection differs significantly from that of chronic infection. During acute infection the immune system is highly activated by virus particles. At the end of the infection, the virus is eliminated, and memory cells have been differentiated. The patient shows acquired immunity. Conversely, in chronic infections the virus is not eliminated and the continuously released virus particles result in T cell exhaustion (Figure 1). It is not yet clear if T cell exhaustion arises during acute infections (29). PD-1 upregulation has been noted in the context of acute viral infection with vaccinia virus, influenza virus, rabies in mice, and hepatitis A virus (HAV), HBV, and HCV in humans (21, 53, 94, 95). During the acute phase of infection, virus-specific T cells respond to activation with increased expression of PD-1 among other markers of activation. Additionally, the virus upregulates PD-L1 on different cell types either directly through pattern recognition receptor signaling or indirectly by prompting the release of inflammatory cytokines (96). Studies that examine the functional role of the PD-1 pathway during acute infection suggest that PD-1 is involved in protecting against lethal immunopathology (37, 97). For example, during the acute phase of infection with the chronic strain of LCMV, PD-1-deficient mice die from lethal immunopathology within 1 week after infection (21, 34). The severe immunopathology is characterized by high levels of systemic cytokines and pulmonary pathology as a result of endothelial cell death (34). In contrast, PD-1-deficient mice infected with the acute strain of LCMV responded similarly to infection when compared to wild type animals (21). These results suggest two important ideas regarding the role of the PD-1 pathway during acute infection. First, they highlight the capacity of certain strains of virus to establish a chronic infection by subverting immune clearance during the acute phase of infection by taking advantage of the immunosuppressive properties of the PD-1 pathway. Second, they underscore the importance of the PD-1 pathway in dampening the immune response during the acute phase of persistent infections to protect against severe immunopathology.

Studies exploring respiratory viral infections in mice using influenza virus (IAV) and human metapneumovirus (HMPV) also support the role for the PD-1 pathway in reducing acute phase immunopathology (64, 98, 99). In these experiments, anti-PD-L1 treatment of mice infected with HMPV resulted in improved pulmonary CD8^+^ T cell function and reduced lung viral titers within 1-week post-infection. Anti-PD-L1 treatment was associated with increased levels of the pro-inflammatory cytokines IFN-γ, TNFα, and IL-6. While exacerbated lung pathology was not observed in mice treated with anti-PD-L1 when compared to mice treated with an isotype control, the mice treated with anti-PD-L1 exhibited a higher degree of airway dysfunction as measured by pulse oximetry (64) (Table 1). PD-1-deficient mice infected with IAV exhibited similar airway dysfunction and recover weight more slowly when compared to wildtype mice (64). Congruent with these data, histological sections of tissue collected from the lower airways of pediatric patients with severe H1N1 influenza A virus reveal increased expression of PD-1 and PD-L1, thereby supporting the role that the expression of PD-1 and its ligand are important in the context of inflammation in human subjects (64).

Acute hepatitis B virus infection can lead to liver cirrhosis, dangerous organ failure and necessary liver transplantation. Studies in human patients examining acute hepatitis B (AHB) virus infection reveal a similar pattern to that observed in the lung tissue of patient's infected with influenza. In a clinical cohort of patients diagnosed with AHB, the authors report that PD-1 expression was significantly upregulated on HBV-specific CD8^+^ T cells during the early phase of acute HBV, and that viral clearance correlated with a subsequent decrease in PD-1 expression. Importantly, patients with acute liver failure expressed lower levels of PD-1 within 1–2 weeks after the clinical onset of disease (53). Thus, dysfunctional PD-1 expression on HBV-specific CD8^+^ T cells positively correlates with liver failure (15). Not only do these data support the PD-1 pathway being an important regulator of tissue damage, but they also suggest that the expression of PD-1 may act as a potential indicator for the clinical outcome of AHB infection. Additionally, these studies also suggest that PD-1-mediated signaling not only attenuates HBV-specific CD8^+^ T cell effector function during the acute phase of infection but also that PD-1 expression early during infection positively correlates with the development of HBV-specific memory CD8^+^ T cells following disease resolution (53).

Given the known role that PD-1 signaling has on tempering T cell responses, therapeutic blockade of the pathway would theoretically augment T cell effector function during acute infection (Table 1). Indeed, therapeutic blockade of the PD-1 pathway during acute infection with the rabies virus enhanced both the immune response and survival rate in a murine model (65). Additional studies exploring murine models of influenza, reveal that checkpoint blockade improves CD8^+^ T cell function by increasing IFN-γ and granzyme B production (64, 66). Murine models of acute infection also support a role for the PD-1 pathway in regulating the development of T memory cells. In the absence of PD-1 signaling, antigen-experienced CD8^+^ T cells taken from mice infected with vaccinia virus preferentially differentiate to central memory CD8^+^ T cells, whereas wild type CD8^+^ T cells preferentially differentiate into the effector memory cells. Additionally, antigen-specific CD8^+^ T cells taken from PD-1-deficient mice produced higher levels of cytokines when compared to antigen-specific CD8^+^ T cells taken from wildtype mice (100).

Interestingly, data derived from patients infected with the Ebola virus suggests that the PD-1 pathway plays an important role in facilitating Ebola-virus-induced immune subversion. CD4^+^ and CD8^+^ T cells taken from patients infected with Ebola virus exhibited markedly increased levels of PD-1 expression, impaired IFN-γ production, and inversion of the CD4/CD8 T cell ratio. Importantly, surviving patients showed significantly lower expression of the PD-1 and the coinhibitory receptor CTLA-4 as well as lower inflammation as measured by pro-inflammatory cytokines (e.g., TNFα, IL-8, MIP-1β, etc.) (101, 102). These observations suggest that PD-1 blockade may help enhance survival of patients infected with Ebola virus.

## PD-1 Pathway in Bacterial and Parasitic Infections

The PD-1 pathway has also been examined in the context of numerous bacterial infections. Persistent infections with bacteria have also been associated with suboptimal T cell responses. For example, the gastrointestinal pathogen, *Helicobactor pylori* establishes chronic infections in humans leading to gastroduodenal ulcers and gastric cancer (103–105). T cell dysfunction and PD-1 expression have been observed in patients infected with *H. pylori* (67, 106, 107). Human gastric epithelial cells taken from patients infected with *H. pylori* express higher levels of PD-L1 when compared to controls (67, 107). PD-L1 upregulation induced by *H. pylori* is mediated by the Sonic Hedgehog signaling pathway (103). This infection-associated PD-L1 expression may help explain the increased frequency of gastric cancer among people infected with *H. pylori*, whereby PD-L1 expression on premalignant lesions permits immune checkpoint evasion thereby facilitating progression toward malignancy (103). Furthermore, *in vitro* co-cultures of human gastric epithelial cell lines infected with *H. pylori* and CD4^+^ T cells demonstrate that treatment with anti-PD-L1 antibodies results in enhanced CD4^+^ T cell proliferation and IL-2 production (67). These data not only support the role that *H. pylori*-induced PD-L1 expression plays in promoting persistent infection, but also implicate *H. pylori*-induced PD-L1 expression on gastric epithelial cells as an important player in immune evasion and cancer progression.

In contrast to these data, PD-1-deficient mice result in decreased survivability of mice infected with *Mycobacterium tuberculosis* (Mbt) (108, 109). PD-1-deficient mice infected with Mbt exhibited uncontrolled bacterial proliferation, focal necrosis, and lower numbers of infiltrating T and B cells. Additionally, the pro-inflammatory cytokines TNFα, IL-1, IL-6, and IL-17A were markedly increased in the lung and sera of PD-1-deficient mice infected with Mbt (109). Interestingly, CD4^+^ T cells exhibit increased and sustained PD-1 expression during Mbt infection in both mice and humans (108–110). However, Mbt-specific CD8^+^ T cell responses in PD-1-deficient mice were normal (108). These data suggest that Mbt acquires a fitness advantage in the absence of PD-1 and that PD-1 expression on CD4^+^ T cells specifically exerts a protective effect against Mbt infection. Indeed, there are reports of Mbt reactivation among patients with cancer being treated with PD-1 blockade (111). Depleting CD4^+^ T cells in PD-1-deficient mice during infection with Mbt rescued the animal's ability to survive infection (108). These data highlight the important role that inhibition through the PD-1 pathway plays in preventing T cell-driven immunopathology and exacerbation of infection with Mbt. These results again support the role of the PD-1 pathway in both shaping the immune response and protecting against severe immunopathology.

Why does PD-1 blockade in *H. pylori* infection enhance the immune response whereas lack of PD-1 in Mbt infection worsens the extent of infection? These seemingly conflicting responses may be partially explained by the type and number of CTLs, T_eff_, and T_ex_ cells present at the site of infection, their ratios to T_reg_ cells, and their degree of PD-1 and costimulatory molecule expression. In the context of infection with *H. pylori*, gastric biopsy samples from infected patients reveal that specimens with higher frequency of both gastric infiltrating T_reg_ cells and PD-1^+^ CD4^+^ T cells correlates directly with the density of *H. pylori*, suggesting that these cell types permit bacterial colonization rather than control it (112). This is in contrast to the T_h_1 effector cells that are required to control intracellular *Mbt* infection (113). Despite being an intracellular pathogen, it has also been demonstrated that CD8^+^ T cells do not recognize Mbt-infected macrophages (114). It follows that targeting the PD-1 pathway in the context of either infection would likely have differential effects owing to the fact that PD-1 blockade would effectively be targeting different cell types. At present, PD-1 blockade as a treatment in bacterial infection is poorly studied and only a limited amount of papers has been published. One possible explanation for the difficulty in defining T cell responses against bacteria is that a single species of bacteria can be commensal, opportunistic, or even pathogenic depending on the host's immune system, location, and route of inoculation. As it follows, T cells must respond to the same organism differently. This inherent complexity may present itself with challenges when attempting assess T cell responses to bacterial infection. Even the bacterial route of entry has been shown to elicit different types of T_eff_ cells, where intravenous inoculation of *Listeria monocytogenes* favors the differentiation of long-lived T_h_1 memory cells whereas intra-nasal inoculation favors shorter-lived T_h_17 cells (115).

While the role of the PD-1 pathway in the context of T cells has namely been studied among a limited number of bacteria, there is a growing body of evidence that supports a role for the PD-1 pathway in directly regulating the response of B lymphocytes and innate immune cells, such as macrophages and dendritic cells. PD-1 expression on B cells has been shown to suppress protective humoral immune responses to *Streptococcus pneumoniae* by inhibiting clonal expansion and IgG production by *S. pneumoniae*-specific B cells (116). On dendritic cells, PD-1 suppresses the immune system's protective responses against infection with *Listeria monocytogenes* (117) and on macrophages, PD-1 expression is associated with macrophage dysfunction and promotes lethality secondary to bacteremia and sepsis (118). These data illustrate the need for more extensive research on the PD-1 pathway in the context of bacterial infection.

T cell exhaustion has been described during infection with a number of parasitic protozoans as well. Chronic infection in context of parasitic infection are common because of the ability of parasites to escape the immune system by increasing anti-inflammatory molecules such as TGF-β and IL-10. In addition, parasitic infections are associated with increased expression of coinhibitory receptors and their ligands (29). For example, PD-1 expression in persistent infections with *T. gondii, L. major*, and *Plasmodium falciparum* (70, 119) have been reported. Mice infected with the intracellular protozoan parasite *T. gondii* exhibit persistent infections and CD8^+^ T cell exhaustion (68). Blocking the PD-1 pathway in mice infected with *T. gondii* reinvigorated CD8^+^ T cell responses and prevented mortality in chronically infected animals (68). Infection with *Leishmania major* can present with different clinical manifestations. Cutaneous leishmaniasis causes local lesions were parasite replication is contained, not unlike the focal lesions observed in granulomatous tuberculosis. In contrast, visceral leishmaniasis is characterized by disseminated infection, immunosuppression, and severe disease. In both canine and murine models of chronic infection with leishmaniasis, CD4^+^ and CD8^+^ T cells express high levels of PD-1 and reduced levels of pro-inflammatory cytokines (120, 121). PD-1 blockade in animals infected with *Leishmania infantum* resulted in enhanced production of IFN-γ and nitric oxide (NO), whereas IL-4 and IL-10 were decreased (69). Blocking PD-L1 in infections with *Leishmania donovani* leads to T cell proliferation and decreased parasite burden (70). Interestingly, there is data that supports a differential role between PD-L1 and PD-L2 in murine parasitic infections. For example, PD-L1-deficient mice were protected from symptoms associated with *Leishmania mexicana* infection whereas PD-L2-deficient mice were not (29). Similarly, increased PD-L2 expression during malaria infection is associated with reduced parasite load and limited T cell exhaustion (29, 122).

## PD-1 Pathway and Sepsis

Of particular interest is the role of the PD-1 pathway during sepsis. Sepsis is a heterogeneous syndrome that generally presents with a hyper-inflammatory state that is then followed by a compensatory anti-inflammatory or immunosuppressive state. There are over 30 million cases and 6 million deaths worldwide per year (123). Of the patients that survive the initial hyper-inflammatory state progress to the immunosuppressive state where they still remain difficult to treat. Indeed, the majority of deaths caused by sepsis occur during this latter phase (124). Impaired immunity during sepsis is now recognized to be caused by systemic levels of T cell exhaustion (125). Early studies of PD-1 expression on macrophages in the context of sepsis were associated with reduced bacterial clearance. This sparked a more thorough investigation of the role that the PD-1 pathway plays in bacteremia. In these experiments, macrophages taken from PD-1-deficient mice were resistant to sepsis-induced cellular dysfunction, as defined by diminished bacteriocidal ability, decreased cytokine production, and suppressed antigen presenting capacity (118). Additionally, PD-1 upregulation on CD4^+^ and CD8^+^ T cells has been observed as early as 48 h after the onset of sepsis in both mouse and human subjects (125–127). PD-1-deficient mice show improved survival during sepsis when compared to their wild type counterparts, thereby suggesting a relationship between PD-1 expression, immune dysfunction, and the pathology associated with this highly acute inflammatory response (118). Similarly, administering blocking antibodies against the PD-1 pathway in septic mice increased their long-term survival (118, 128).

While PD-1 expression on T cells during sepsis is well-established, it is not clear whether T cells are the major therapeutic targets of PD-1 blockade during septic immune dysfunction. Understanding the role that the PD-1 pathway plays in sepsis is important for the proper selection of patients for treatment with immunomodulatory drugs. Given these results, a number of clinical trials are underway to investigate PD-1 blockade and a growing body of research is quickly developing around the role of the PD-1 pathway in sepsis.

## Conclusion

It is clear that the PD-1 pathway plays an important role during chronic infection with numerous pathogens. As mentioned above, the PD-1 pathway permits pathogens to escape elimination by the host's immune system and thereby establish chronic infection. While inhibiting this pathway in some models of infection clearly reinvigorates T_ex_ cells, not all models of PD-1 blockade elicit this response. More work is needed to understand the role of additional coinhibitory receptors in the context of infection. Involvement multiple coinhibitory pathways may explain why blocking the PD-1 pathway in the context of some infections is sufficient to restore T_ex_ cell function while in others it is not. By better describing T_ex_ cells in the context of acute and chronic infections with different pathogens, we may be able to more carefully employ combination therapies aimed at blocking two or more coinhibitory pathways. Indeed, combination therapy has already been successfully employed to treat mice with chronic malaria (129). Indeed, given the promising results seen in humanized mice infected with HIV and primates infected with SIV, investigating the impact that combining ART with blocking antibodies against the PD-1 pathway is of significance.

While the role of the PD-1 pathway during acute infection is less well-defined, there is evidence to suggest that its expression can act as an important biomarker to predict disease outcomes. Using PD-1 expression as a marker for immune cell activation or immune cell dysfunction during acute (and chronic infection) may have advantages when considering treatment options and the cost of care for patients with difficult to treat microbial infections. Furthermore, PD-1 expression on T cells, B cells, and monocytes has been associated with severe immune dysfunction, an example of which is the second phase of sepsis. A broader understanding of the role that the PD-1 pathway plays on both innate and adaptive immune cells may help us better understand the immune dysregulation observed during sepsis. Interestingly, the immune dysregulation observed during the latter phase of sepsis resembles the acute immune dysfunction observed during infection with the Ebola virus. Drawing parallels between these two inflammatory states may help investigators piece together the pathways circumvented during sepsis or infection with the Ebola virus. While rapid translation of blocking antibodies against the PD-1 pathway from bench to clinic has been observed over the past decade, the role of PD-1 blockade for the treatment of infectious disease is still not clear and it requires further investigation. It is important that we better define the role that the PD-1 pathway has in the context of acute and chronic infection as well as during severe immune dysregulation. Not only might PD-1 expression act as an important biomarker to predict disease outcomes or optimize care for patients with difficult to treat infections, but evaluating the efficacy of blocking the PD-1 pathway in the context of difficult to treat infections may add to our arsenal of drugs to fight drug-resistant pathogens.

## Author Contributions

All authors contributed to conception and design of the study. JJ, ZB, and FS researched and wrote the content of the review article. All authors contributed to manuscript revision, read and approved the submitted version.

### Conflict of Interest

The authors declare that the research was conducted in the absence of any commercial or financial relationships that could be construed as a potential conflict of interest.
